# ﻿First contribution to *Labiobaetis* Novikova & Kluge in Cambodia (Ephemeroptera, Baetidae), with description of two new species

**DOI:** 10.3897/zookeys.1123.90308

**Published:** 2022-10-04

**Authors:** Thomas Kaltenbach, Jhoana Garces, Jean-Luc Gattolliat

**Affiliations:** 1 Museum of Zoology, Palais de Rumine, Place Riponne 6, CH-1005 Lausanne, Switzerland Museum of Zoology Lausanne Switzerland; 2 University of Lausanne (UNIL), Department of Ecology and Evolution, CH-1015 Lausanne, Switzerland University of Lausanne Lausanne Switzerland; 3 Ateneo Biodiversity Research Laboratory, Department of Biology, School of Science and Engineering, Ateneo de Manila University, Quezon City, 1108 Metro Manila, Philippines Ateneo de Manila University Quezon Philippines

**Keywords:** COI, genetic distance, integrated taxonomy, Southeast Asia

## Abstract

Material collected in 2018 in Cambodia gives us first insights into the diversity of *Labiobaetis* Novikova & Kluge, 1987 in this country. No species has been reported so far. We identified two new species using a combination of morphology and genetic distance (COI, Kimura 2-parameter). They are described and illustrated based on their larvae. A key to all *Labiobaetis* species of continental Southeast Asia is provided. The interspecific K2P distance between the two new species is 20–21%, the intraspecific distance of one of them is 1%. The total number of *Labiobaetis* species worldwide is augmented to 156.

## ﻿Introduction

The genus *Labiobaetis* Novikova & Kluge, 1987 (Novikova and Kluge 1987) is one of the richest genera of mayflies with 154 previously described species (Barber-James et al. 2013; Kaltenbach and Gattolliat 2021; Sivaruban et al. 2022). The distribution of *Labiobaetis* is nearly worldwide, exept for the Neotropical realm, New Zealand, and New Caledonia; its main diversity is found in Southeast Asia (Kaltenbach and Gattolliat 2019, 2020, 2021; Kaltenbach et al. 2020) and New Guinea (Kaltenbach and Gattolliat 2018, 2021; Kaltenbach et al. 2021). The history and concept of the genus *Labiobaetis* were recently summarized in detail (Shi and Tong 2014; Kaltenbach and Gattolliat 2018). Together with *Pseudopannota* Waltz & McCafferty, 1987, it belongs to the tribe Labiobaetini, established by Kluge and Novikova (2016) based on a unique combination of imaginal and larval characters. *Labiobaetis* is part of Baetidae, the family with the highest species diversity among mayflies, comprising over 1160 species in 118 genera (Sartori and Brittain 2015; Jacobus et al. 2019; updated), which is approximately one-third of all mayfly species worldwide.

In the past years, the diversity of *Labiobaetis* in Southeast Asia was intensely studied with focus on the archipelagos of Indonesia (including the whole of Borneo) and the Philippines (Kaltenbach and Gattolliat 2019, 2020, 2021; Kaltenbach et al. 2020). Many new species were described based on morphological and molecular evidence. This contribution will shift our focus to continental Southeast Asia, starting with a first contribution to the knowledge of *Labiobaetis* in Cambodia. Further studies of the genus in the region are in preparation.

Cambodia is located in the southern part of the Indochinese Peninsula in Southeast Asia, bordering Laos in the northwest, Thailand in the north and the east, and Vietnam in the south and the west, and with a long coastline along the Gulf of Thailand in the west. It is geographically characterized by large central wetlands around Tonle Sap Lake, and by the upper reaches of the Mekong River delta towards Vietnam, surrounded by uplands and low mountains. Cambodia’s rich biodiversity is based on its seasonal tropical rainforests.

So far, the specific diversity of *Labiobaetis* and of Baetidae in general in Cambodia was unknown, despite a first study on mayflies including the first general report of the genus in the country (Chhorn et al. 2020). Some work was done in the neighbouring Vietnam, including a key for the identification of Ephemeroptera (Soldán 1991; Mekong River Commission 2006), and several studies on Baetidae were recently done in the neighbouring Thailand (e.g. Kluge and Suttinun 2020; Suttinun et al. 2020, 2021, 2022). Intensive exchange between these faunas is likely, as there are only rather low mountain chains with large corridors inbetween, and no other barriers between them. In China, an important study on *Labiobaetis* was done by Shi and Tong (2014). In the present study, we describe two new species of *Labiobaetis* from Cambodia based on larval stage.

## ﻿Materials and methods

Materials used in the study were obtained as part of the Cambodia Entomology Initiative aquatic insect ecological study expeditions (Freitag et al. 2018; Chhorn et al. 2020). The specimens were preserved in 96% ethanol.

Dissection of larvae was done in Cellosolve (2-Ethoxyethanol) with subsequent mounting on slides with Euparal liquid, using an Olympus SZX7 stereomicroscope.

The DNA of part of the specimens was extracted using non-destructive methods allowing subsequent morphological analysis (see Vuataz et al. 2011 for details). We amplified a 658 bp fragment of the mitochondrial gene cytochrome oxidase subunit 1 (COI) using the primers LCO 1490 and HCO 2198 (Folmer et al. 1994; see Kaltenbach and Gattolliat 2020 for details). Sequencing was done with Sanger’s method (Sanger et al. 1977). The genetic variability between specimens was estimated using Kimura-2-parameter distances (K2P; Kimura 1980), calculated with the program MEGA 7 (Kumar et al. 2016; http://www.megasoftware.net).

GenBank accession numbers are given in the sections of examined material.

Drawings were made with an Olympus BX43 microscope. To facilitate the determination of species and the comparison of important structures, we partly use a combination of dorsal and ventral aspects in one drawing. Explanations are given in Kaltenbach et al. (2020: fig. 1).

**Figure 1.**
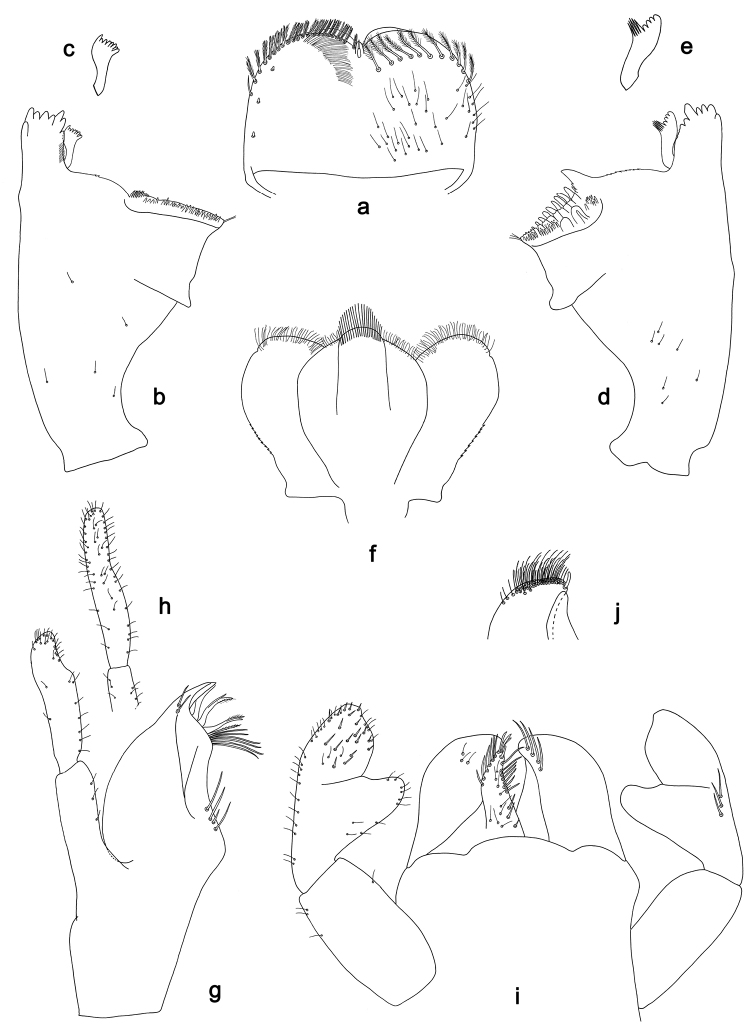
**a–g, i, j***Labiobaetisbrao* sp. nov., larva morphology **a** labrum (left: ventral view, right: dorsal view) **b** right mandible **c** right prostheca **d** left mandible **e** left prostheca **f** hypopharynx and superlinguae**g** maxilla **i** labium (left: ventral view, right: dorsal view) **j** apex of paraglossa **h***Labiobaetisparaoperosus*: maxillary palp.

Photographs of larvae were taken using a Canon EOS 6D camera and processed with the programs Adobe Photoshop Lightroom (http://www.adobe.com) and Helicon Focus v. 5.3 (http://www.heliconsoft.com). Photographs were subsequently enhanced with Adobe Photoshop Elements 13.

The distribution maps were generated with the program SimpleMappr (https://simplemappr.net; Shorthouse 2010).

The dichotomous key was elaborated with the support of the program DKey v. 1.3.0 (http://drawwing.org/dkey; Tofilski 2018).

The terminology follows Hubbard (1995) and Kluge (2004).

### ﻿Abbreviations

**RUPP**Cambodia Entomology Initiative, Royal University of Phonm Phen (RUPP), temporarily stored in Ateneo de Manila University, Quezon City, Philippines (AdMU);

**MZL** Musée de Zoologie Lausanne (Switzerland).

## ﻿Results

### ﻿Definition of groups and description of their characters

*Labiobaetisoperosus* group (*L.brao* sp. nov.) and *sumigarensis* group (*L.kui* sp. nov.) were defined and characterized in Kaltenbach and Gattolliat (2019) and Kaltenbach et al. (2020).

#### ﻿ Labiobaetis brao sp. nov.

Taxon classificationAnimaliaEphemeropteraBaetidae

1D2BF28F-1C4B-5EE0-914C-52BFEF043B43

https://zoobank.org/A5F61492-39FC-4FBC-A77F-C5620E5EDC1E

Figs 1 , 2 , 6b

##### Diagnosis.

**Figure 2.**
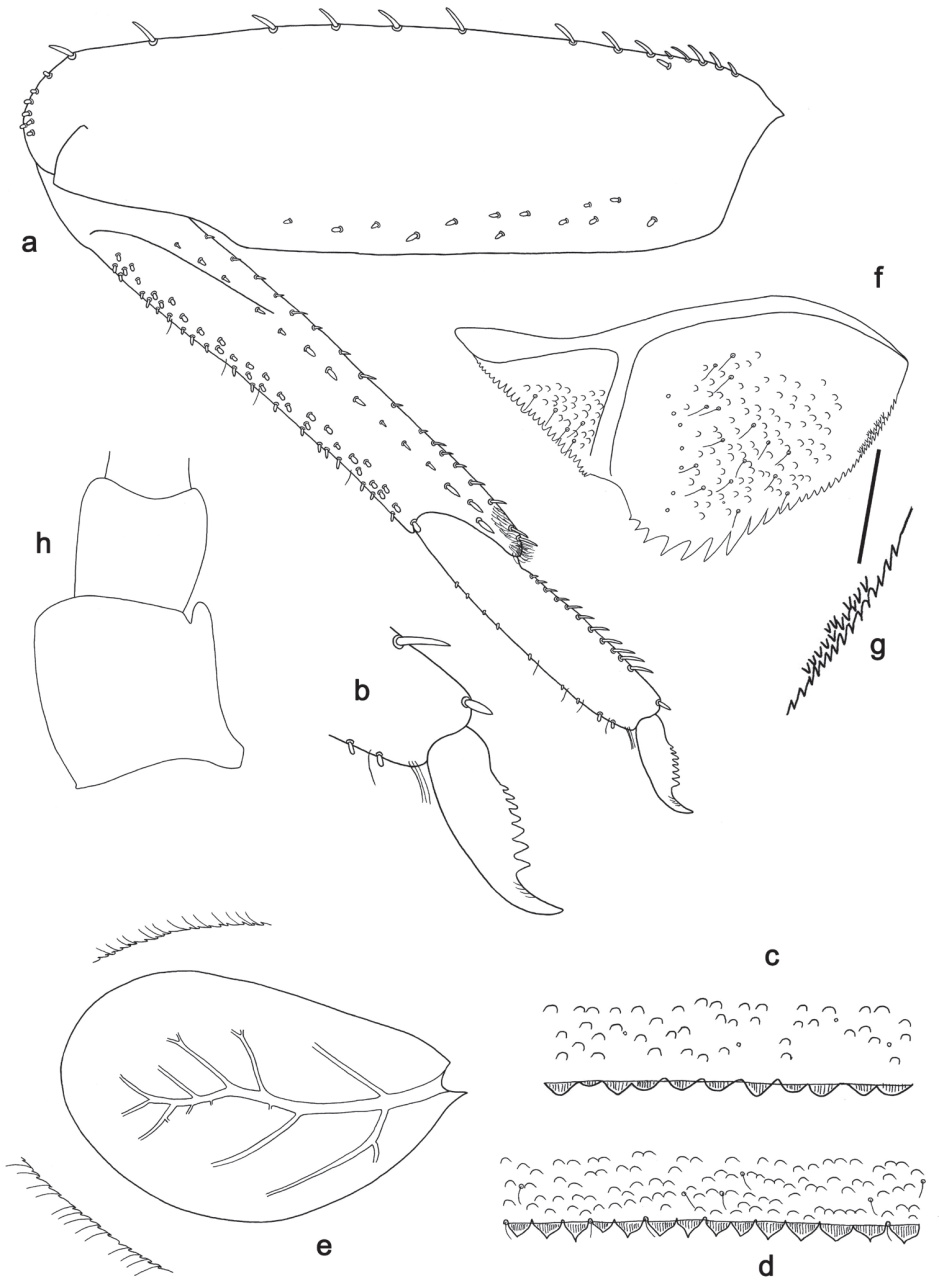
**a–c, e–h***Labiobaetisbrao* sp. nov., larva morphology **a** foreleg **b** fore claw **c** tergum IV **e** tergalius IV **f** paraproct **g** spines along paraproct margin **h** antennal base **d***Labiobaetisparaoperosus*: tergum IV.

**Larva.** Following combination of characters: A) antennal scape with well developed distolateral process (Fig. 2g); B) dorsal surface of labrum with submarginal arc of 9–11 feathered setae (Fig. 1a); B) labial palp segment II with broad, extended, thumb-like distomedial protuberance; segment III rather oblong, apically truncate (Fig. 1h); C) fore femur rather broad, length ca 3× maximum width, dorsal margin with 11–25 curved, short, spine-like setae (Fig. 2a); D) hind protoptera well developed; E) seven pairs of tergalii; F) paraproct distally not expanded, with ca 34 marginal spines and additional row of minute spines along inner, proximal margin (Fig. 2e, f).

##### Description.

**Larva** (Figs 1a–g, i, j, 2 a–c, e–h, 5a, b). Body length 6.4–8.4 mm. Cerci: ca 2/3 of body length. Paracercus: ca 2/3 of cerci length. Antenna: approximately twice as long as head length.

***Colouration*** (Fig. 5a, b). Head, thorax and abdomen dorsally grey-brown, with pattern as in Figure 6a. Abdominal tergits I and X brighter. Fore protoptera light grey-brown with dark striation. Head ventrally brownish, thorax and abdomen ventrally light grey-brown. Legs ecru to light brown, femur with grey-brown distomedial spot, apex and dorsal margin grey-brown. Caudalii grey-brown.

***Antenna*** (Fig. 2h) with scape and pedicel subcylindrical, with well-developed distolateral process at scape.

***Labrum*** (Fig. 1a). Subrectangular, length 0.65× maximum width. Distal margin with medial emargination and a small process. Dorsally with medium, fine, simple setae scattered over surface; submarginal arc of setae composed of 9–11 long, feathered setae. Ventrally with marginal row of setae composed of lateral and anterolateral long, feathered setae and medial long, bifid, pectinate setae; ventral surface with ca three short, spine-like setae near lateral and anterolateral margin.

***Right mandible*** (Fig. 1b, c). Incisor and kinetodontium fused. Incisor with five denticles; kinetodontium with three denticles, inner margin of innermost denticle with a row of thin setae. Prostheca robust, apically denticulate. Margin between prostheca and mola slightly convex, with few minute denticles. Tuft of setae at apex of mola present.

***Left mandible*** (Fig. 1d, e). Incisor and kinetodontium fused. Incisor with four denticles; kinetodontium with three denticles. Prostheca robust, apically with small denticles and comb-shaped structure. Margin between prostheca and mola slightly convex, with minute denticles. Tuft of setae at apex of mola present.

Both mandibles with lateral margins almost straight. Basal half with fine, simple setae scattered over dorsal surface.

***Hypopharynx and superlinguae*** (Fig. 1f). Lingua approx. as long as superlinguae. Lingua longer than broad; medial tuft of stout setae well developed, broad; distal half laterally expanded. Superlinguae distally rounded; lateral margin rounded; fine, long, simple setae along distal margin.

***Maxilla*** (Fig. 1g). Galea-lacinia ventrally with two simple, apical setae under canines. Inner dorsal row of setae with three denti-setae, distal denti-seta tooth-like, middle and proximal denti-setae slender, bifid and pectinate. Medially with one spine-like seta and three long, simple setae. Maxillary palp 1.3× as long as length of galea-lacinia; 2-segmented; palp segment II approximately as long as segment I; setae on maxillary palp fine, simple, scattered over surface of segments I and II; apex of last segment rounded, with excavation at inner distolateral margin.

***Labium*** (Fig. 1i, j). Glossa basally broad, narrowing toward apex; shorter than paraglossa; inner margin with 8–10 spine-like setae, distalmost seta much longer than other setae; apex with three medium and one short, robust setae; outer margin with ca 6 spine-like setae; ventral surface with fine, simple, scattered setae. Paraglossa subrectangular, curved inward; apex rounded; with three rows of long, robust, distally pectinate setae in apical area and three medium, simple setae in anteromedial area; dorsally with a row of four long, spine-like, simple setae near inner margin. Labial palp with segment I 0.8× length of segments II and III combined. Segment I ventrally with short, fine, simple setae. Segment II with broad, extended, thumb-like distomedial protuberance; distomedial protuberance 0.7× width of base of segment III; ventral surface with short, fine, simple setae; dorsally with a row of three long, spine-like setae near outer margin. Segment III rather oblong, apically truncate; length 1.1× width; ventrally covered with short, spine-like, simple setae and short, fine, simple setae.

***Hind protoptera*** well developed.

***Foreleg*** (Fig. 2a, b). Ratio of foreleg segments 1.4:1.0:0.6:0.2. ***Femur*.** Length ca 3× maximum width. Dorsal margin with 11–25 curved, short, spine-like setae, often one seta additionally near margin in basal area; length of setae 0.14× maximum width of femur. Apex rounded, with a spine-like seta and some short, stout setae. Many stout, lanceolate setae scattered along ventral margin; femoral patch absent. ***Tibia*.** Dorsal margin with row of short, stout, apically rounded setae, and some fine, simple setae; many more stout, apically rounded setae along dorsal margin; on apex one seta of same type. Ventral margin with row of short, curved, spine-like setae, on apex some longer setae and a tuft of fine, simple setae. Anterior surface with row of stout, lanceolate setae near ventral margin. Patellatibial suture present on basal 1/3 area. ***Tarsus*.** Dorsal margin with row of short, stout setae and some fine, simple setae. Ventral margin with row of curved, spine-like setae. Claw with one row of 7–10 denticles; distally pointed; with ca four stripes; subapical setae absent.

***Middle and hind legs*.** As foreleg, but with reduced or rudimentary femoral patch on middle femur, and reduced or well developed on hind femur.

***Terga*** (Fig. 2c). Surface with irregular rows of U-shaped scale bases. Posterior margin of tergum IV with spines varying between mostly triangular to mostly rounded, wider than long.

***Tergalii*** (Fig. 2e). Present on segments I–VII. Margins with small denticles intercalating fine simple setae. Tracheae extending from main trunk to inner and outer margins. Tergalius I ca 2/3 length of segment II. Tergalius IV as long as length of segments V and 1/2 VI combined. Tergalius VII as long as length of segment VIII.

***Paraproct*** (Fig. 2f, g). Distally not expanded, with ca 34 stout, marginal spines, and additional row of minute spines along inner, proximal margin. Surface scattered with U-shaped scale bases and fine, simple setae. Cercotractor with numerous small, marginal spines.

##### Etymology.

The new species is dedicated to the indigenous Brao people from northeastern Cambodia.

##### Distribution.

Cambodia (Fig. 6b).

##### Biological aspects.

The specimens were mainly collected in secondary forest remnants at altitudes of 100 m, partly on littoral gravel.

##### Type material.

***Holotype*.** Cambodia • larva; Kampong Speu Province, Kokie waterfall, sec. forest remnants; 110 m; 11°12'11"N, 104°03'49"E; 12.07.2018; leg. H. Freitag and J. Garces; on slide; GBIFCH00592700; MZL. ***Paratypes*.** Cambodia • 8 larvae; same data as holotype; 1 on slide; GenBank ON982739; GBIFCH00829878; RUPP; 1 on slide; GBIFCH00975576; MZL; 6 in alcohol; GBIFCH00975580, GBIFCH00975581; MZL • 1 larva; Kampong Speu Province, Chambok River, 1.83 Km from Chambok Community, sec. forest, littoral gravel; 240 m; 11°21'58"N, 104°06'17"E; 11.07.2018; leg. H. Freitag and J. Garces; on slide; GBIFCH00592730; RUPP.

#### ﻿ Labiobaetis kui sp. nov.

Taxon classificationAnimaliaEphemeropteraBaetidae

62089C43-D7B3-5583-8483-1625E704FD8A

https://zoobank.org/03B09E8B-57E2-40AA-8BAD-911A6D969606

**Figure 3.**
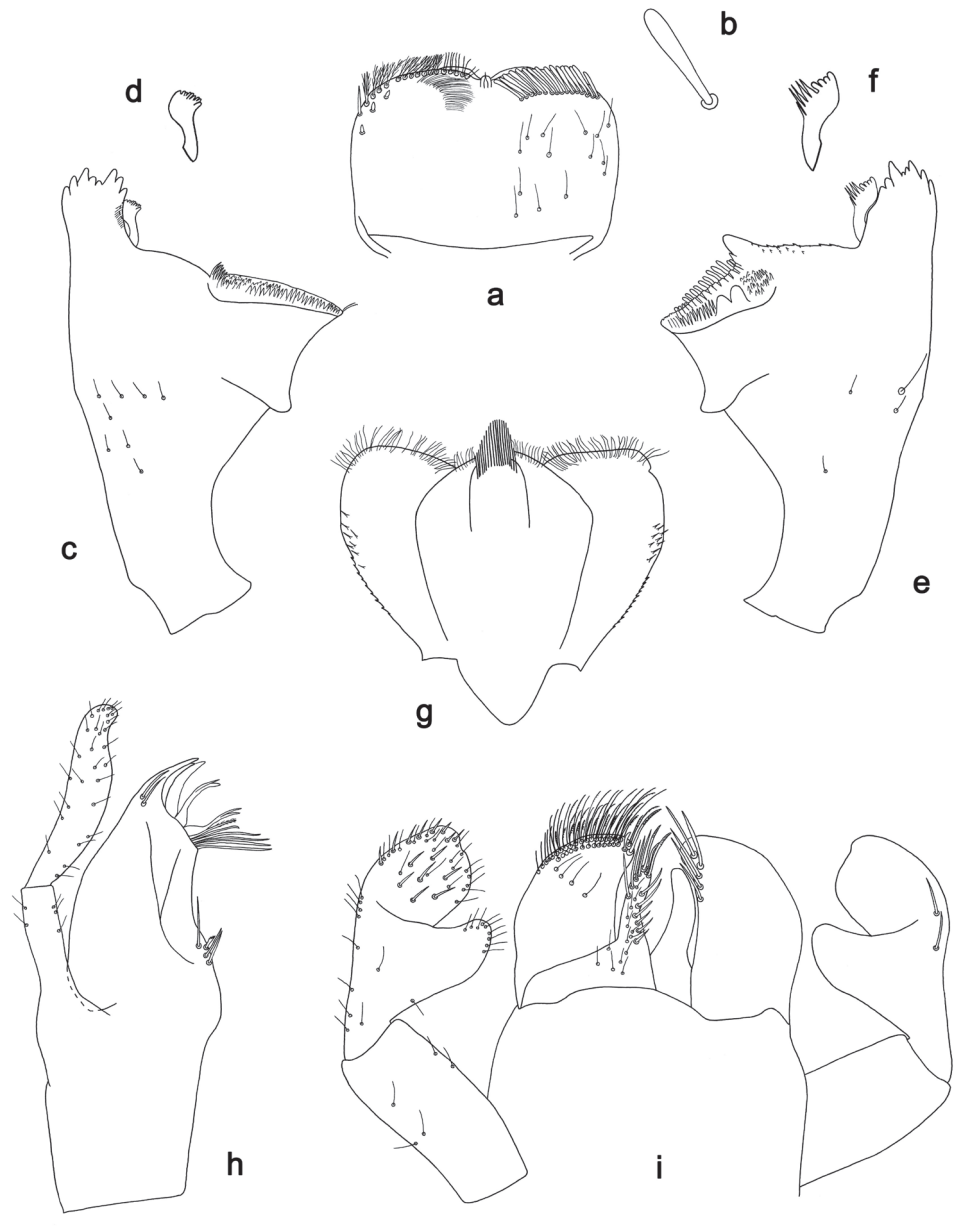
*Labiobaetiskui* sp. nov., larva morphology **a** labrum (left: ventral view, right: dorsal view) **b** seta of submarginal arc **c** right mandible **d** right prostheca **e** left mandible **f** left prostheca **g** hypopharynx and superlinguae **h** maxilla **i** labium (left: ventral view, right: dorsal view).

**Figure 4.**
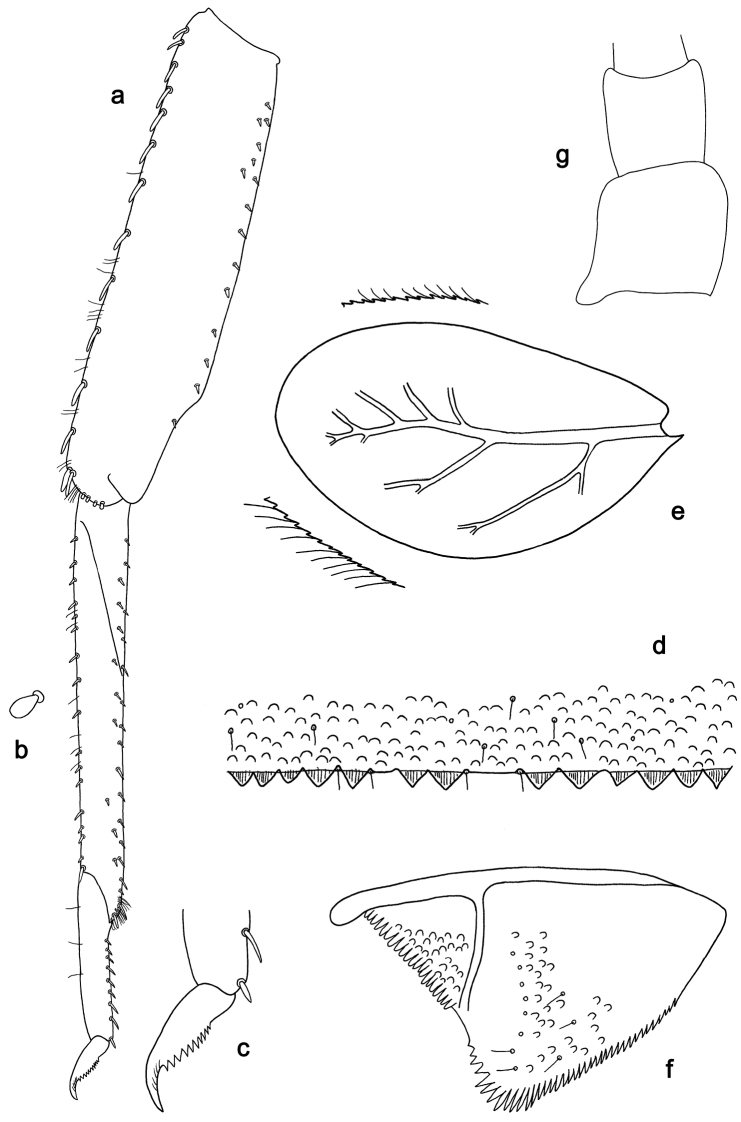
*Labiobaetiskui* sp. nov., larva morphology **a** foreleg **b** seta of tibia dorsal margin **c** fore claw **d** tergum IV **e** tergalius IV **f** paraproct **g** antennal base.

**Figure 5.**
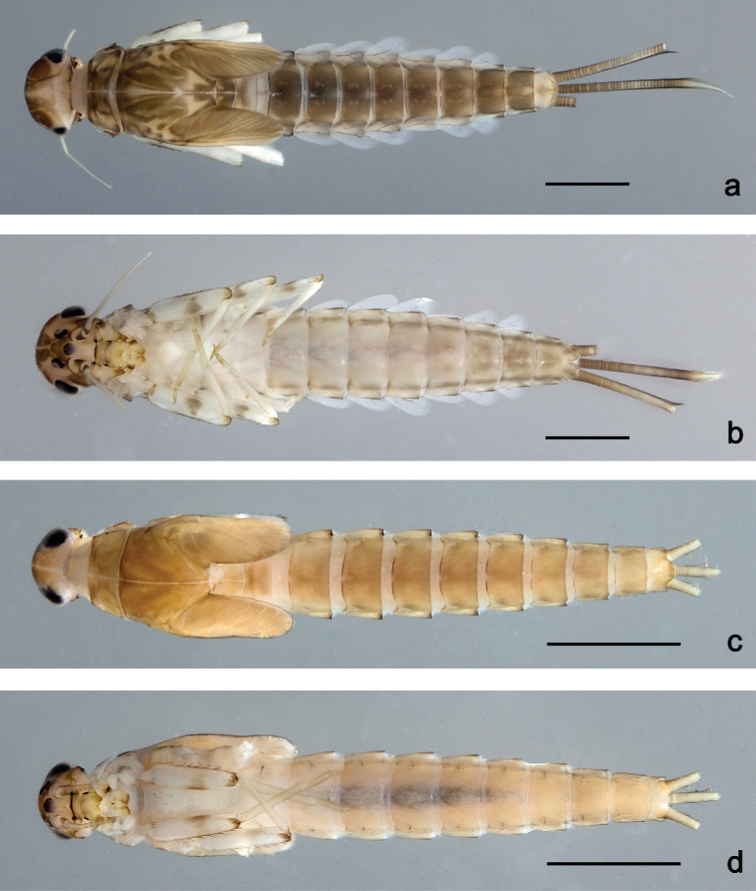
Habitus, larvae **a***Labiobaetisbrao* sp. nov., dorsal view **b***Labiobaetisbrao* sp. nov., ventral view **c***Labiobaetiskui* sp. nov., dorsal view **d***Labiobaetiskui* sp. nov., ventral view. Scale bar: 1 mm.

**Figure 6.**
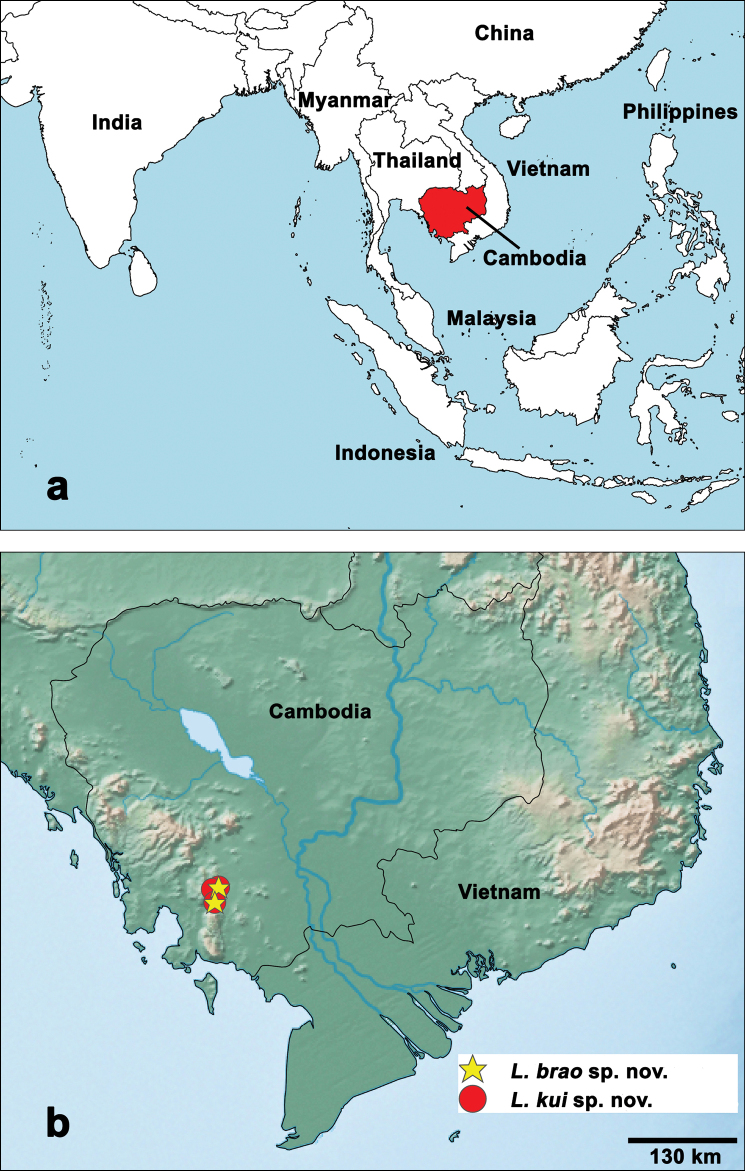
Distribution of *Labiobaetis* in Cambodia **a** overview map **b***Labiobaetis* species.

Figs 3 , 4 , 5c, d , 6b

##### Diagnosis.

**Larva.** Following combination of characters: A) antennal scape without process (Fig. 4g); B) dorsal surface of labrum with submarginal arc of 16–18 long, clavate setae (Fig. 3a); C) labial palp segment II with an extended, slightly hooked, thumb-like distomedial protuberance (Fig. 3i); D) left mandible without setae at apex of mola (Fig. 3e); E) fore femur rather slender, length ca 4× maximum width, dorsal margin with 10–15 curved, spine-like setae (Fig. 4a); F) hind protoptera absent; G) six pairs of terga­lii; H) paraproct distally slightly expanded, with 33–38 stout, marginal spines (Fig. 4f).

##### Description.

**Larva** (Figs 3, 4, 5c, d). Body length ca 4.9 mm. Caudalii broken. Antenna broken.

***Colouration*** (Fig. 5c, d). Head, thorax, and abdomen dorsally uniform brown. Head, thorax, and abdomen ventrally light brown. Legs light brown; femur with a brown medial spot, darker on ventral margin, dorsal margin and apex brown. Caudalii light brown.

***Antenna*** (Fig. 4g) with scape and pedicel subcylindrical, without distolateral process at scape.

***Labrum*** (Fig. 3a, b). Rectangular, length 0.7× maximum width. Distal margin with medial emargination and a small process. Dorsally with medium, fine, simple setae scattered over surface; submarginal arc of setae composed of 16–18 long, clavate setae. Ventrally with marginal row of setae composed of anterolateral long, feathered setae and medial long, bifid setae; ventral surface with ca three short, spine-like setae near lateral and anterolateral margin.

***Right mandible*** (Fig. 3c, d). Incisor and kinetodontium fused. Incisor with five denticles; kinetodontium with three denticles, inner margin of innermost denticle with a row of thin setae. Prostheca robust, apically denticulate. Margin between prostheca and mola slightly convex. Tuft of setae at apex of mola present.

***Left mandible*** (Fig. 3e, f). Incisor and kinetodontium fused. Incisor with five denticles; kinetodontium with three denticles. Prostheca robust, apically with small denticles and comb-shaped structure. Margin between prostheca and mola straight, with minute denticles towards subtriangular process. Tuft of setae at apex of mola absent.

Both mandibles with lateral margins almost straight. Basal half with fine, simple setae scattered over dorsal surface.

***Hypopharynx and superlinguae*** (Fig. 3g). Lingua approx. as long as superlinguae. Lingua longer than broad; medial tuft of stout setae well developed; distal half laterally expanded. Superlinguae distally almost straight; lateral margin rounded; fine, long, simple setae along distal margin.

***Maxilla*** (Fig. 3h). Galea-lacinia ventrally with two simple, apical setae under canines. Inner dorsal row of setae with three denti-setae, distal denti-seta tooth-like, middle and proximal denti-setae slender, bifid. Medially with one pectinate, spine-like seta and three or four medium, simple setae. Maxillary palp 1.2× as long as length of galea-lacinia; 2-segmented; palp segment II 1.2× length of segment I; setae on maxillary palp fine, simple, scattered over surface of segments I and II; apex of last segment rounded, with excavation at inner distolateral margin.

***Labium*** (Fig. 3i). Glossa basally broad, narrowing toward apex; shorter than paraglossa; inner margin with ca six spine-like setae increasing in length distally; apex with two long and one medium, robust, pectinate setae; outer margin with ca four spine-like setae; ventral surface with fine, simple, scattered setae. Paraglossa subrectangular, curved inward; apex rounded; with three rows of long, robust, distally pectinate setae in apical area and a row of 2–4 medium, simple setae in anteromedial area; dorsally with a row of four or five long, spine-like, simple setae near inner margin. Labial palp with segment I 0.8× length of segments II and III combined. Segment I ventrally with short, fine, simple setae. Segment II with extended, slightly hooked, thumb-like distomedial protuberance; distomedial protuberance 0.7× width of base of segment III; ventral surface with short, fine, simple setae; dorsally with two long, spine-like, simple setae near outer margin. Segment III slightly pentagonal; apex rounded, inner apical margin slightly concave; length subequal to width; ventrally covered with short, spine-like, simple setae and short, fine, simple setae.

***Hind protoptera*** absent.

***Foreleg*** (Fig. 4a–c). Ratio of foreleg segments 1.3:1.0:0.4:0.2. ***Femur*.** Length ca 4× maximum width. Dorsal margin with 10–15 long, curved, spine-like setae; length of setae 0.23× maximum width of femur. Apex rounded, with a pair of long, curved, spine-like setae and some short, stout setae. Many stout, lanceolate setae scattered along ventral margin; femoral patch absent. ***Tibia*.** Dorsal margin with row of short, stout, apically rounded setae, on apex one longer, spine-like seta. Ventral margin with row of short, curved, spine-like setae, on apex some longer setae and a tuft of fine, simple setae. Anterior surface scattered with stout, lanceolate setae near ventral margin. Patellatibial suture present on basal 1/3 area. ***Tarsus*.** Dorsal margin with some fine, simple setae. Ventral margin with row of curved, spine-like setae. Claw with one row of nine or ten denticles; distally pointed; with ca. five stripes; subapical setae absent.

***Terga*** (Fig. 4d). Surface with irregular rows of U-shaped scale bases and scattered fine, simple setae. Posterior margin of tergum IV with triangular spines, wider than long.

***Tergalii*** (Fig. 4e). Present on segments II–VII. Margins with small denticles intercalating fine simple setae. Tracheae extending from main trunk to inner and outer margins. Tergalius IV as long as length of segments V and 1/3 VI combined. Tergalius VII as long as length of segment VIII.

***Paraproct*** (Fig. 4f). Distally slightly expanded, with 33–38 stout, marginal spines. Surface scattered with U-shaped scale bases, fine, simple setae and micropores. Cercotractor with numerous small, marginal spines.

##### Etymology.

The new species is dedicated to the indigenous Kui people from northeastern Cambodia.

##### Distribution.

Cambodia (Fig. 6b).

##### Biological aspects.

The specimens were collected from 100 m to 640 m, mostly on littoral gravel.

##### Type material.

***Holotype*.** Cambodia • larva; Kampong Speu Province, Chambok River, 1.83 Km from Chambok Community; 240 m; 11°21'58"N, 104°06'17"E; 11.07.2018; leg. H. Freitag and J. Garces; on slide; GBIFCH00592702; MZL. ***Paratypes*.** Cambodia • 14 larvae; same data as holotype; 1 on slide; GBIFCH00592701; MZL; 13 in alcohol; GenBank ON982737, ON982738; GBIFCH00515681, GBIFCH00829876, GBIFCH00829877, GBIFCH00975577, GBIFCH00975578; MZL • 7 larvae; Kampong Speu Province, waterfall at Kirirom National Park; 640 m; 11°20'26"N, 104°02'14"E; 13.07.2018; leg. H. Freitag and J. Garces; 1 on slide; GBIFCH00592698; RUPP; 6 in alcohol; GBIFCH00975579; MZL • 1 larva; Kampong Speu Province, Kokie waterfall, secondary forest remnants; 110 m; 11°12'11"N, 104°03'49"E; 12.07.2018; leg. H. Freitag and J. Garces; on slide; GBIFCH00592699; RUPP.

### ﻿Key to *Labiobaetis* species of continental Southeast Asia (larvae)

**Table:** 

1	Setae of submarginal arc dorsally on labrum simple, pointed (Kaltenbach et al. 2020: fig. 2a)	**2**
–	Setae of submarginal arc dorsally on labrum feathered or clavate (clavate setae apically pectinate or smooth) (Figs 1a, 3a, b; Shi and Tong 2014: fig. 7)	**4**
2	Right mandible with pronounced hump between prostheca and mola (Shi and Tong 2014: fig. 24)	***L.numeratus* (Müller-Liebenau, 1984)**
–	Right mandible without hump between prostheca and mola	**3**
3	Tergalii present on abdominal segments I-VII; hind protoptera well developed (Müller-Liebenau 1984: fig. 9i); femoral patch present	***L.multus* (Müller-Liebenau, 1984)**
–	Tergalii present on abdominal segments II-VII; hind protoptera minute (Müller-Liebenau 1984: fig. 10i); femoral patch absent	***L.moriharai* (Müller-Liebenau, 1984)**
4	Setae of submarginal arc dorsally on labrum feathered (Fig. 1a)	**5**
–	Setae of submarginal arc dorsally on labrum clavate (apically smooth or pectinate) (Fig. 3a, b)	**7**
5	Hind protoptera absent	***L.difficilis* (Müller-Liebenau, 1984)**
–	Hind protoptera present, well developed (Müller-Liebenau 1984: fig. 8i)	**6**
6	Distomedial protuberance of labial palp segment II slightly curved upwards (Fig. 1i); paraproct with additional rows of minute spines at distal margin (Fig. 2f, g)	***L.brao* sp. nov.**
–	Distomedial protuberance of labial palp segment II slightly curved downwards (Müller-Liebenau 1984: fig. 8g); paraproct without extra rows of spines (Müller-Liebenau 1984: fig. 8l)	***L.operosus* (Müller-Liebenau, 1984)**
7	Hind protoptera present, well developed (Shi and Tong 2014: fig. 5)	***L.ancoralis* Shi & Tong, 2014**
–	Hind protoptera absent	**8**
8	Antennal scape with slightly developed distolateral process (Müller-Liebenau 1984: fig. 6f); tarsus ventrally with row of feathered, spine-like setae; posterior margin of tergite IV with triangular spines, apically sharply pointed (Müller-Liebenau 1984: fig. 39)	***L.diffundus* (Müller-Liebenau, 1984)**
–	Antennal scape without distolateral process (Fig. 4g); tarsus ventrally with row of spine-like setae (not feathered); posterior margin of tergite IV with triangular spines, apically mostly blunt (Fig. 4d)	***L.kui* sp. nov.**

### ﻿Genetics

COI sequences were obtained from both new species (see type material sections). The genetic distance (K2P) between them is 20–21%, and therefore much higher than 3.5%, which is generally considered as a likely maximal value for intraspecific divergence (Hebert et al. 2003; Ball et al. 2005). A very limited genetic distance of 1% was found between two specimens of *L.kui* sp. nov., as expected for the same location.

## ﻿Discussion

### ﻿Assignment to *Labiobaetis* and to species groups

For the assignment of the new species to *Labiobaetis* we refer to Kluge and Novikova (2014), Müller-Liebenau (1984), and McCafferty and Waltz (1995). *Labiobaetis* is characterized by a number of characters, some of which are not found in other taxa (Kluge and Novikova 2014): antennal scape sometimes with a distolateral process (Fig. 2h); maxillary palp two segmented with excavation at inner distolateral margin of segment II, excavation may be poorly developed or absent (Figs 1g, 3h); labium with paraglossae widened and glossae diminished; labial palp segment II with distomedial protuberance (Figs 1i, 3i). All these characters vary and may be secondarily lost (Kluge and Novikova 2014). The concept of *Labiobaetis* is also based on additional characters, summarized and discussed by Kaltenbach and Gattolliat (2018, 2019).

The morphological groups within *Labiobaetis* are primarily a working tool but could also serve as a basis for future studies on the generic or subgeneric delimitations and phylogeny of this genus. The inclusion of nuclear gene sequences may prove that some of them are natural groups. The two species in Cambodia belong to different groups, one to the *operosus* goup and one to the *sumigarensis* group. The *operosus* group is mainly characterized by A) labrum dorsally with submarginal arc of feathered setae; B) distolateral process at scape well developed; C) seven pairs of tergalii; D) hind protoptera well developed (see Kaltenbach et al. 2020: 40). The *sumigarensis* group is mainly characterized by A) labrum dorsally with submarginal arc of clavate setae; B) left mandible without setae at mola apex; C) six pairs of tergalii; D) hind protoptera absent; E) colour dorsally uniform brown (see Kaltenbach et al. 2020: 63).

These groups are widespread and highly diversified in Asia. Species of the *operosus* group are also known from India, Malaysia, Indonesia, and the Philippines; and species of the *sumigarensis* group from India, Sri Lanka, Malaysia, Indonesia, Brunei, China, Taiwan, and the Philippines (Müller-Liebenau 1984; Müller-Liebenau and Hubbard 1985; Kang et al. 1994; Shi and Tong 2014; Kubendran et al. 2015; Kaltenbach and Gattolliat 2019, 2020; Kaltenbach et al. 2020; Sivaruban et al. 2022). None of these groups are known from New Guinea (Kaltenbach and Gattolliat 2018, 2021; Kaltenbach et al. 2021).

Apart from *Labiobaetisbrao* sp. nov. (*operosus* group), there is another species of this group in continental Southeast Asia, *L.operosus* (Müller-Liebenau, 1984). *Labiobaetisbrao* sp. nov. is different from *L.operosus* by a labial palp segment II protuberance slightly directed distad (slightly directed proximad in *operosus*, Müller-Liebenau 1984: fig. 8g) and by minute additional spines along the inner proximal margin of the paraproct (Fig. 2g; absent in *operosus*, Kaltenbach and Gattolliat 2019: fig. 35d). The most similar species to *L.brao* sp. nov. is *L.paraoperosus* Kaltenbach & Gattolliat from Sumatra. It is different by a maxillary palp with slight distolateral excavation (Fig. 1h; strong excavation in *L.brao* sp. nov.); labial palp segment II with thumb-like protuberance very broad, not narrowing toward apex (Kaltenbach and Gattolliat 2019: fig. 36h; less broad and narrowing toward apex in *L.brao* sp. nov.); spines at posterior margin of tergite IV triangular, pointed (Fig. 2d; rounded spines in *L.brao* sp. nov.). Apart from *L.kui* sp. nov., there is also another species of group *sumigarensis* in continental Southeast Asia, *L.diffundus* (Müller-Liebenau, 1984). *Labiobaetiskui* sp. nov. is different by the absence of a distolateral process at antennal scape (Fig. 4g; small process in *diffundus*, Müller-Liebenau 1984: fig. 6f), by a labial palp segment II protuberance relatively narrow with distinctly rounded apex (Fig. 3i; broader with less rounded apex in diffundus, Müller-Liebenau 1984: fig. 6g), and by spines at proximal margin of tergum IV much wider than long (Fig. 4d; slightly wider than long in *diffundus*, Müller-Liebenau 1984: fig. 39).

### ﻿Genetic distance

The genetic distances between the two new species of *Labiobaetis* in the Cambodia (20–21%, K2P) is rather high, which is in line with the genetic distances found in Indonesia (11–24%; Kaltenbach and Gattolliat 2019), Borneo (19–25%; Kaltenbach and Gattolliat 2020), and the Philippines (15–27%; Kaltenbach et al. 2020). Ball et al. (2005) reported a mean interspecific, congeneric distance of 18% for mayflies from the United States and Canada.

The number of sampled localities and different habitats is until now very limited and the vast majority of the country was not covered by collection activities so far (Fig. 6b). Therefore, we can expect that the number of *Labiobaetis* species in Cambodia will substantially increase with further collections.

## Supplementary Material

XML Treatment for Labiobaetis brao

XML Treatment for Labiobaetis kui
